# Quartz-enhanced multiheterodyne resonant photoacoustic spectroscopy

**DOI:** 10.1038/s41377-024-01425-1

**Published:** 2024-03-22

**Authors:** Jiapeng Wang, Hongpeng Wu, Angelo Sampaolo, Pietro Patimisco, Vincenzo Spagnolo, Suotang Jia, Lei Dong

**Affiliations:** 1 State Key Laboratory of Quantum Optics and Quantum Optics Devices, Institute of Laser Spectroscopy, Shanxi University, Taiyuan, 030006 China; 2 Collaborative Innovation Center of Extreme Optics, Shanxi University, Taiyuan, 030006 China; 3 PolySense Lab, Dipartimento Interateneo di Fisica, University and Politecnico of Bari, CNR-IFN, Via Amendola 173, 70126 Bari, Italy

**Keywords:** Optical sensors, Photoacoustics, Optical spectroscopy, Frequency combs

## Abstract

The extension of dual-comb spectroscopy (DCS) to all wavelengths of light along with its ability to provide ultra-large dynamic range and ultra-high spectral resolution, renders it extremely useful for a diverse array of applications in physics, chemistry, atmospheric science, space science, as well as medical applications. In this work, we report on an innovative technique of quartz-enhanced multiheterodyne resonant photoacoustic spectroscopy (QEMR-PAS), in which the beat frequency response from a dual comb is frequency down-converted into the audio frequency domain. In this way, gas molecules act as an optical-acoustic converter through the photoacoustic effect, generating heterodyne sound waves. Unlike conventional DCS, where the light wave is detected by a wavelength-dependent photoreceiver, QEMR-PAS employs a quartz tuning fork (QTF) as a high-*Q* sound transducer and works in conjunction with a phase-sensitive detector to extract the resonant sound component from the multiple heterodyne acoustic tones, resulting in a straightforward and low-cost hardware configuration. This novel QEMR-PAS technique enables wavelength-independent DCS detection for gas sensing, providing an unprecedented dynamic range of 63 dB, a remarkable spectral resolution of 43 MHz (or ~0.3 pm), and a prominent noise equivalent absorption of 5.99 × 10^-6 ^cm^-1^·Hz^-1/2^.

## Abstract (web summary)

We introduce an innovative quartz-enhanced multiheterodyne resonant photoacoustic spectroscopy technique that utilizes a quartz tuning fork to achieve resonant detection of a dual comb based on the photoacoustic effect.

## Graphical abstract

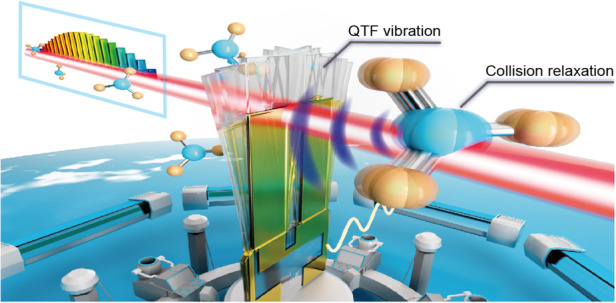

## Introduction

Dual-comb spectroscopy (DCS) is an emerging spectroscopic tool that combines the benefits of tunable laser absorption spectroscopy with the main characteristics of conventional broadband spectroscopy into a single platform^1–3^. It has been considered as an attractive tool with a significant impact in diverse fields. For example, DCS has been applied to ultra-broadband infrared (IR) spectroscopy^4,5^, near-field microscopy for subwavelength spatial resolution^6,7^, high-precision metrology of molecular line centers^8,9^, digital holography^10^, nonlinear spectroscopy^11^, and greenhouse gas monitoring^12^. As DCS continues to mature, the list of proof-of-concept experiments and applications will continue to expand.

DCS generally down-converts the optical response signals of two optical frequency combs to the radio frequency heterodyne signals through the beat note pairs of the dual-comb, thus overcoming the photoreceiver’s inability to react immediately to changes in light field strength, and the limitation in a single comb’s resolution imposed by a dispersive spectrometer. Moreover, DCS accomplishes Fourier transform spectroscopy without mechanical scanning^3,13,14^. However, the challenges of using DCS to measure broadband spectroscopy were clear from very early on. First, the dynamic range of a DCS-based spectrometer is restricted by its single photoreceiver since it can only withstand limited optical power^1,15^. With a fixed optical power, the distributed power of each comb tooth becomes lower as the number of teeth increases, resulting in a low signal-to-noise ratio (SNR). In addition, a photoreceiver receives all noise within its detection bandwidth, deteriorating its ultimate performance. Secondly, the spectral resolution of a DCS-based spectrometer is determined by the comb mode linewidth as well as the time window during which the temporal waveform is measured^4,16^. For gas-phase molecular spectroscopy in the near-infrared spectroscopy (NIR) spectral region, the linewidth of a pressure-broadened spectral line is within the range of 1–5 GHz. However, the linewidth of a Doppler-broadened spectral line at room temperature is within the range of 300–900 MHz. In Doppler-free spectroscopy, heavy molecules or cold samples have an even narrower linewidth^17,18^. Ideally, the comb tooth spacing (the repetition frequency) should be similar to the desired spectral resolution. Hence, the tooth spacing must be adjustable over a wide range to interrogate all these spectral lines. On the other hand, extending the time window size to match narrower comb tooth spacing can increase the noise contribution and acquisition time when the majority of the signal components are observed. Thirdly, so far, although the spectral span of DCS has been able to cover 14 octaves across the terahertz (THz), infrared (IR), and visible range^19–21^, commercially available photoreceivers used to access different spectral regions, are based on different material systems, such as silicon (400–1100 nm), InGaAs (800–1600 nm), and HgCdTe (3–10 μm)^22^. It means that an optical frequency comb in a specific spectral region requires an appropriate photoreceiver. A Fourier-Transform based optical frequency comb photoacoustic spectroscopy approach is reported to achieve high-resolution Fourier-transform spectroscopy without the need for a photoreceiver^23^. However, compared to DCS-based technology, there is still potential for further advancements in terms of mechanical scanning-free and rapid measurements.

In total, 20 Hz to 20 kHz is the commonly referenced audio frequency range, having slower temporal dynamics than the typical vibrational-translational relaxation rates of gas molecules. Thus, gas molecules can be used as converters of the electromagnetic intensity changes into sound waves through the photoacoustic effect when the optical signals are frequency down-converted to the audio frequency domain. A sound transducer such as a microphone can then be employed, together with gas molecules, to form a dual-comb detector based on optical-to-acoustic-to-electric energy conversion. The advantage of this photoacoustic method is its wavelength independence, therefore making it applicable in dual-comb measurements^24,25^ ranging from the ultraviolet (UV) to the mid-infrared (MIR), even to the terahertz (THz), without detector switching. However, similar to the limitations imposed by a single photoreceiver in conventional DCS, the use of a wideband microphone imposes restrictions on the dynamic range and the ultimate spectral resolution, due to the simultaneous sampling of all photoacoustic signals and the time window of the Fourier transform.

Recently, quartz tuning fork (QTF), originally invented as a frequency standard for electronic clocks, has been widely used as sound wave detectors in photoacoustic spectroscopy^26–29^. The QTF is a piezo-electric element that converts its deformation into the separation of electrical charges, enabling it to detect weak acoustic pressure waves generated between its two prongs, where optical radiation interacts with a trace gas. Acoustically, the QTF possesses a quadrupole geometry that makes it selectively sensitive to the sound originating in a small space between its prongs. Sound waves from distant acoustic sources tend to move the QTF prongs in the same direction, thus resulting in no electrical response^30^. Such characteristics determine its excellent environmental noise immunity and the simplest configuration. The QTF is mass-produced at very low cost ( < 1$) and has been proven to operate over a wide temperature range from 1.56 K (superfluid helium) to 700 K^31^. The QTF has a small footprint, allowing the realization of ultra-compact detection modules for analyzing gas samples down to a few mm^3^ in volume^30,32^.

The combination of QTF and DCS in the audio frequency domain gives rise to a new technique of quartz-enhanced multiheterodyne resonant photoacoustic spectroscopy (QEMR-PAS), which has several advantages over the photoreceiver/microphone-based DCS for detecting multiheterodyne signals. The QTF is a sharply resonant acoustic transducer with extremely low internal losses, causing a super-high Q factor ranging from 10^4^ to 10^5^. Hence, the acoustic energy can be accumulated in the QTF, dispensing with additional sonic enhancement elements, such as multi-pass cells^22,33^. The super-high Q factor also provides a detection bandwidth of 0.3–3 Hz in the audio frequency domain, enabling high-performance acoustic filtering during optical-to-acoustic energy conversion. Moreover, as a resonant acoustic transducer, the QTF picks up the resonance signal from the multiheterodyne signals, thus offering a super-large linear dynamic range from the thermal noise to the QTF’s breakdown deformation, corresponding to the sound intensity level from –17 dB to 46 dB (see Supplementary Note S1), covering six orders of magnitude. However, it should be noted that a recent approach has been reported where all the comb lines are compressed into the sharp resonance profile of a QTF, followed by Fourier transform^34^. This method is still based on the conventional DCS scheme, but the QTF is an ultra-narrow bandwidth transducer, unlike a wideband photoreceiver/microphone. Consequently, the number of comb teeth that can be utilized is limited by the QTF’s narrow bandwidth. Moreover, the resolution requirements brought by dense comb teeth also impose higher demands on the coherent time.

Different from the conventional DCS method, QEMR-PAS extracts the frequency component that resonated with the QTF from the multiple heterodyne sound tones every time, thus removing the time-window restriction imposed by the Fourier transform method. Hence, QEMR-PAS has a lower mutual-coherence requirement for two combs. In terms of the device, QEMR-PAS employs a simple phase-sensitive detector for the QTF signal processing instead of a complex Fourier transform instrument. The phase-sensitive detector acting as an electrical filter commonly has a <1 Hz electrical bandwidth during acoustic-to-electric energy conversion. The simultaneous realization of the super-narrow bandwidths in acoustics and electronics enables the high spectral resolution of QEMR-PAS.

In this manuscript, we provide a proof-of-concept of the QEMR-PAS technique. The beat frequency response from a dual comb is down-converted to the audio frequency domain. The energy levels of gas molecules are used as optical-acoustic converters, generating multiheterodyne sound signals. A QTF is used to “listen” to the sound resonant with its fundamental flexural mode from the multiheterodyne signals and convert it into a current signal. With this approach, a large-dynamic-range, high-resolution, broad-bandwidth dual-comb spectrometer is achieved within a small footprint.

## Results

### Illustration of concept

**Fig. 1:**
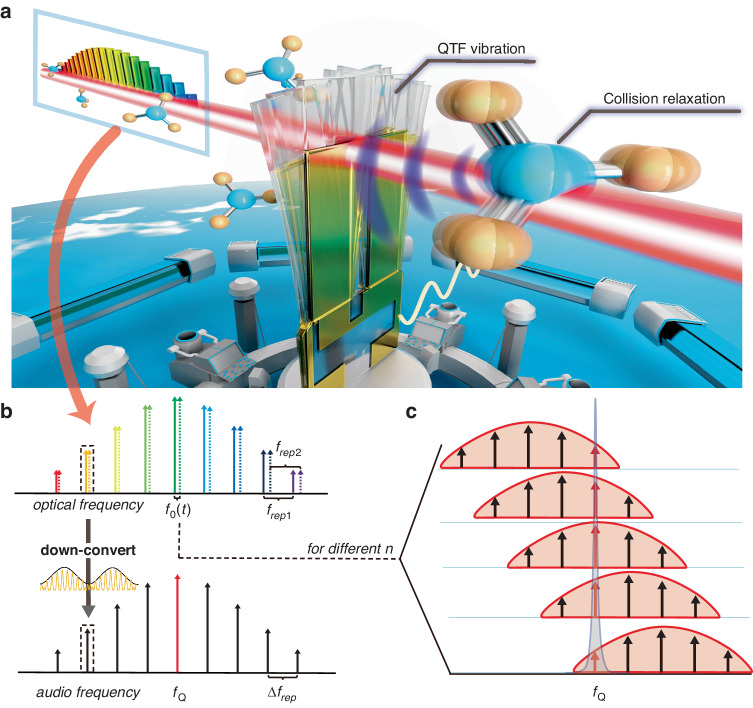
Illustration of QEMR-PAS. **a** Dual comb interacts with gas molecules and generates multiple heterodyne acoustic tones. The acoustic tone resonant with the QTF is enhanced and then converted into an electrical output by the QTF. **b** Conversion from an optical dual comb (on top) to an audio frequency comb (on bottom). An example of down-conversion is demonstrated (in the middle) where the carrier represents the optical frequency, and the envelope represents the modulation frequency in optical power. The red comb line in the audio frequency comb corresponds to the resonance frequency of the QTF. **c** QEMR-PAS utilizes a QTF to detect the sound wave generated by the photoacoustic effect. The red teeth in the audio frequency combs represent the components within the QTF response bandwidth at the different ${f}_{0}(t)$, and the gray shadow is the area of the QTF frequency response

The QEMR-PAS concept is illustrated in Fig. 1. A QTF is immersed in target gas, and a dual comb passes between two prongs of the QTF, as shown in Fig. 1a. The optical frequency components ${f}_{n,1}(\text{t})$ and ${f}_{n,2}(\text{t})$ of two optical frequency combs in QEMR-PAS are given by

1 $${f}_{n,1}\left(t\right)=n{f}_{{rep},1}+{f}_{0,1}\left(t\right)+{f}_{\text{cw}}$$

2 $${f}_{n,2}=n{f}_{{rep},2}+{f}_{0,2}+{f}_{\text{cw}}$$

where ${f}_{{rep},1}$ and ${f}_{{rep},2}$ represent the repetition rates of the two combs, respectively. ${f}_{\text{cw}}$ is the optical carrier frequency, which is identical for the two combs when they are generated from the same laser source. The optical frequency shift of the combs is determined by ${f}_{\mathrm{0,2}}$ and ${f}_{\mathrm{0,1}}(t)$, with ${f}_{\mathrm{0,1}}(t)$ being variable. The subscript 1 and 2 identify the two combs, and $n=0,\,\pm 1,\,\pm 2,\ldots$ represents the modulation order of electro-optic modulation and the comb line indices. It is assumed that ${f}_{{rep},1} \,>\, {f}_{{rep},2}$ and ${f}_{\mathrm{0,1}}(t) \,>\, {f}_{\mathrm{0,2}}$. When two combs are optically combined, this can be interpreted as a single frequency comb, as shown in Fig. 1b, with a modulation frequency in optical power

3 $${f}_{n}(t)={f}_{n,1}(t)-{f}_{n,2}=n({f}_{{rep},1}-{f}_{{rep},2})+{f}_{0,1}(t)-{f}_{0,2}$$

4 $$=n\times \varDelta {f}_{{rep}}+{f}_{0}(t)$$

It is worth noting that the difference in the repetition rates and the optical frequency shift of the two combs is set very small, i.e., $\varDelta {f}_{{rep}}\ll {f}_{{rep},1}\approx {f}_{{rep},2}$, ${f}_{0}(t)\ll {f}_{\mathrm{0,1}}(t)\approx {f}_{\mathrm{0,2}}$, so that the beat frequencies fall in the audio frequency domain, producing an audio frequency comb. After this audio frequency comb interacts with the target gas through the photoacoustic effect, multiple heterodyne sound waves are generated. The sound waves can be detected by the QTF only when the following condition is satisfied

5 $${f}_{n}(t)={f}_{Q}$$

where ${f}_{Q}$ is the resonance frequency of the fundamental flexural mode of the QTF. The value of ${f}_{0}(t)$ for the sequence *n* can be obtained by combining Eq. (4) and Eq. (5).

6 $${f}_{0}(t)={f}_{Q}-n\times \varDelta {f}_{{rep}}$$

Then ${f}_{\mathrm{0,1}}(t)$ must be adjusted to satisfy Eq. (6) for different teeth in succession (as the index *n* increases), as schematically depicted in Fig. 1c. As a result, the photoacoustic signal generated by each tooth of the audio frequency comb is detected by the QTF in turn, and the entire photoacoustic spectrum can be reconstructed.

### Setup

**Fig. 2:**
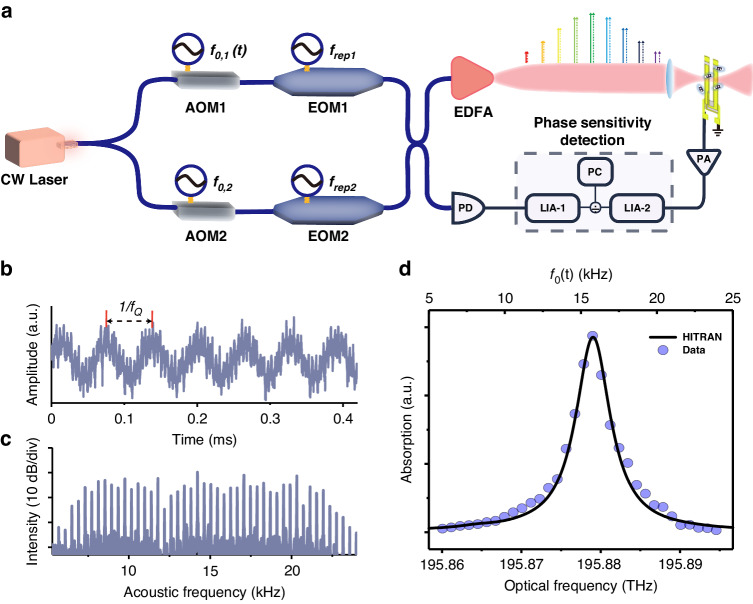
Schematic and results of the QEMR-PAS setup. **a** Experimental setup of QEMR-PAS. A continuous-wave (CW) laser is split into two beams. Each beam is modulated by an acousto-optic modulator (AOM) and an electro-optic modulator (EOM). 99% of the combined light is amplified by an erbium-doped fiber amplifier (EDFA), and the rest (1%) is sent to a photoreceiver. A lock-in amplifier (LIA-1) records the comb intensity and phase fluctuations. The signal from a QTF is amplified by a pre-amplifier (PA) and demodulated by a lock-in amplifier (LIA-2). The data is acquired by a personal computer (PC). **b** Piezoelectric signal from the QTF. It has a sinusoidal wave shape since the QTF just measures the resonant sound wave from the multiple heterodyne sound waves. **c** Audio frequency comb generated through the down-conversion of the optical dual comb. The audio frequency comb is obtained by a spectrum analyzer using the output signal of the photoreceiver. **d** Normalized absorption spectrum of acetylene gas acquired by QEMR-PAS (light blue dots) and contrast with the Hitran model (solid black line) at normal pressure and temperature

The experimental apparatus of QEMR-PAS is depicted in Fig. 2a. A continuous-wave (CW) laser beam is split into two parts, which are frequency shifted by ${f}_{\mathrm{0,1}}(t)$ and ${f}_{\mathrm{0,2}}$, respectively, using two acousto-optic modulators (AOM). Each beam is transmitted through an electro-optic modulator (EOM) to generate an optical frequency comb with a repetition frequency ${f}_{{rep},1}$ and ${f}_{{rep},2}$. Then the two beams recombine to produce a dual comb. 99% of the dual-comb power is introduced into an erbium-doped fiber amplifier (EDFA) and a gas chamber, while the rest reaches a photoreceiver as a reference signal. In the gas chamber, target molecules are excited by the dual comb to a high-energy state, then generating multiple heterodyne sound waves through the molecular periodic collision relaxation process. A custom QTF (see Supplementary Note S2) is used to detect the resonant sound wave from the multiple heterodyne sound tones. The generated piezo-electric current is sent to a transimpedance pre-amplifier (PA) to be converted into a voltage signal. A quadrature lock-in amplifier (LIA-2) is used to demodulate the PA output signal at the QTF resonance frequency. The output of the photoreceiver is sent to another quadrature lock-in amplifier (LIA-1) as a reference signal, whose amplitude and phase are used to normalize the signal and to suppress the phase fluctuation of the system due to the asymmetry between the optical paths of two combs (see Supplementary Note S3).

### Measurements

To provide a proof-of-concept of the QEMR-PAS technique, a 1% C_2_H_2_:N_2_ mixture is used as the target sample to detect the C_2_H_2_ P(11) line of the ${\nu }_{1}+{\nu }_{3}$ absorption band. A narrow linewidth whispering gallery mode laser with an output power of 10 mW is used as the CW laser. Figure 2b shows the PA output signal in the time domain. The sinusoidal-like waveform indicates the excellent capability of the QTF for filtering out the resonant sound signals thanks to its ultra-narrow bandwidth. As a supplement, the audio frequency comb from the photoreceiver output is mapped in the frequency domain, as shown in Fig. 2c. Considering the response time $\tau$ of the QTF ($\tau =\frac{Q}{\pi {f}_{Q}}\approx 300\,{\rm{ms}}$), the integration time of LIA-2 was set to 300 ms, with an acquisition time of 2 seconds for each tooth. Since the 32 comb teeth used in our experiment, the total acquisition time was 64 seconds. It is worth noting that the signal-to-noise ratio (SNR) is only related to the integration time rather than the acquisition time, as the phase-sensitive method is employed instead of Fourier transform.

The setting of ${f}_{0}(t)$ determines which part of the dual comb is selected. For the optical detection of the analytes, the selected spectral range can vary from one tooth to the whole comb. For demonstration, the 32 comb teeth adjacent to the dominant frequency (*n* = 0) were selected. The total optical power of the dual comb is 320 mW, which is too high to be detected by a single-photoreceiver-based dual-comb spectrometer. The compression factor between acoustic and optical frequency axes is given by ${f}_{{rep},1}/\varDelta {f}_{{rep}}\approx 2\times 1{0}^{6}$. The acquired photoacoustic spectrum is shown in Fig. 2d, normalized to the initial tooth intensity signal recorded by LIA-1.

**Fig. 3:**
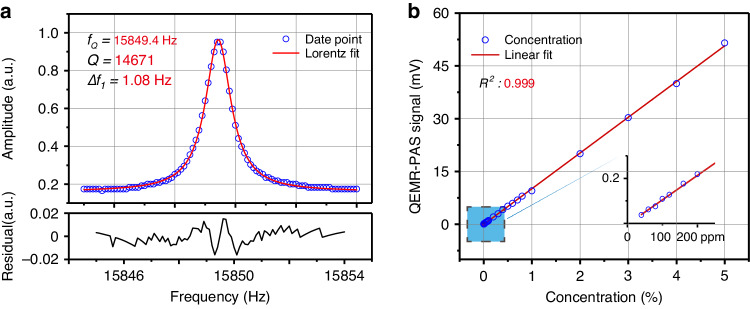
Dynamic range of QEMR-PAS. **a** Frequency response curve of the custom QTF. **b** QEMR-PAS signal as a function of gas concentration. The acetylene concentration levels vary from 40 ppm to 5%. The inset on the right demonstrates the QEMR-PAS linearity at low concentrations from, 40 ppm to 200 ppm

To assess the dynamic range of the system, the response curve of the custom QTF as a function of the excitation frequency is measured, as shown in Fig. 3a. The ultra-narrowband filtering capability ($\varDelta {f}_{1}\approx 1\text{Hz}$) is observed around the QTF’s resonance frequency of 15849.4 Hz, resulting in a *Q* factor of 15000. Moreover, the QTF exhibits an excellent linear response at its resonance frequency from thermal noise level (2 μV) to nearly breakdown voltage (~5 V), which has been experimentally verified (see Supplementary Note S1). The result indicates that the dynamic range of the QTF for sound intensity measurement has a wide span of 63 dB. Subsequently, a concentration measurement experiment is carried out. The QEMR-PAS peak signals of the C_2_H_2_ P(11) line are displayed in Fig. 3b as a function of the C_2_H_2_ concentration level, which successfully demonstrates the excellent linear relationship between the gas concentration and QEMR-PAS signal amplitude (R^2^ value of 0.999). The QEMR-PAS signals for the low concentration levels from 40 ppm-200 ppm are shown in the inset of Fig. 3b. The SNR is defined as the ratio of the mean value of the peak signals to the 1σ value of their standard deviation. At the lowest concentration of 40 ppm, an SNR of ~10 was estimated, corresponding to a detection limit of 4 ppm. Therefore, the linear dynamic range of the system exceeds 40 dB, which is restricted by the gas concentration levels achieved during the experiment. There is still a dynamic reserve of 20 dB to receive the stronger sound signal.

**Fig. 4:**
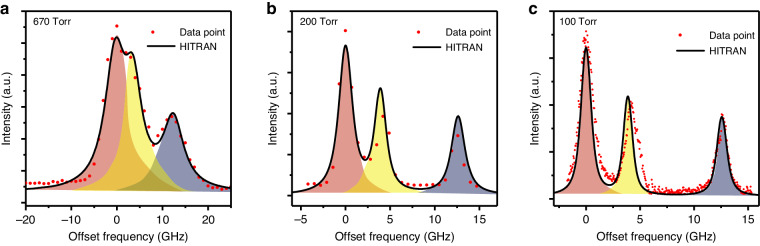
QEMR-PAS spectra of ammonia measured at different spectral resolutions and pressures. **a** Spectral resolution of 1 GHz at the pressure of 670 Torr. Three spectral features overlap each other. **b** Spectral resolution of 700 MHz at the pressure of 200 Torr. The third feature completely separates from the first two. **c** Spectral resolution of 43 MHz at the pressure of 100 Torr. The three features are well separated. The horizontal axis represents the offset frequency from 195.7368 THz (1531.68 nm)

The spectral resolution of a reconstructed spectrum is one of the most important parameters for spectroscopic analysis. An overlapping spectrum of ammonia (NH_3_) consisting of three discrete features near 1531.68 nm is measured to evaluate the QEMR-PAS spectral resolution, as shown in Fig. 4. It is noteworthy that, with an increase in pressure, the photoacoustic signal exhibits an initial rise followed by a subsequent decline. Since we are focusing on resolution, the photoacoustic signal is normalized to the highest value in Fig. 4. The whispering gallery mode laser emitting around 1531 nm is employed as the light source with an ultra-narrow laser linewidth of a few Hz, which can be utilized to study the ultimate limit to the spectral resolution of QEMR-PAS. The spectrum of NH_3_ is reconstructed by the QEMR-PAS system at 670 Torr (Fig. 4a), 200 Torr (Fig. 4b) and 100 Torr (Fig. 4c). With the pressure decreasing, the three NH_3_ absorption features gradually separate as a result of the linewidth narrowing of each feature. In Fig. 4a, the spectral resolution of the system is set to 1 GHz, which is directly determined by the comb spacing. At 200 Torr (Fig. 4b), the comb spacing of the system is set to 700 MHz. At this pressure, the C_2_H_2_ absorption feature peaking at the higher frequency completely separates from the other two. At 100 Torr, the three features are well separated from each other, with the spectral resolution set to 43 MHz (Fig. 4c). The QEMR-PAS system exhibits the ability to distinguish the ammonia absorption lines at low pressures due to the ultra-narrow bandwidth of the QTF. In theory, the QEMR-PAS spectral resolution is just limited by laser linewidth. Higher spectral resolution (meaningful for spectral reconstruction of Doppler-free absorption features) has not been tested.

**Fig. 5:**
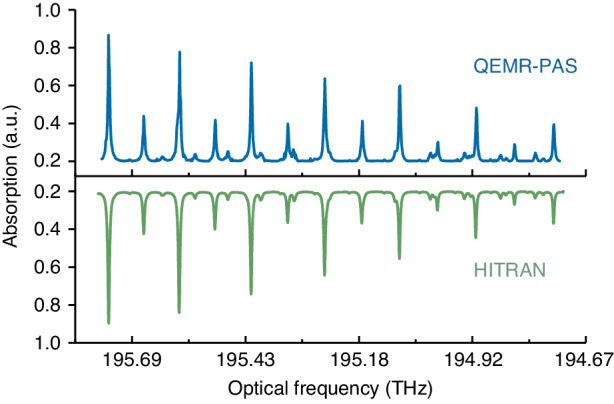
QEMR-PAS and simulated spectra of acetylene from 194.73 THz to 195.76 THz. The blue curve on the top is the measured spectra using QEMR-PAS, and the green curve on the bottom is the simulated spectra using the Hitran database

The employed optical frequency comb has 32 comb teeth evenly arranged on both sides of the dominant frequency with a comb spacing of 1 GHz. Therefore, the frequency comb can cover a frequency span of 31 GHz, significantly larger than the typical mode-hop-free tuning ranges of NIR diode lasers. If more dominant frequencies are provided, a broadband spectrogram with high spectral resolution can be achieved. Hence, an external cavity diode laser (ECDL) is employed. By tuning the ECDL frequency in a step-wise manner, the dual-comb spans nearly 1 THz. For a 1% C_2_H_2_:N_2_ mixture, each QEMR-PAS measurement is carried out with a spectral resolution of 1 GHz. The spectral stitching is applied to obtain the entire spectrogram, as shown in Fig. 5. The P-branch spectral lines in ${\nu }_{1}+{\nu }_{3}$ band of C_2_H_2_ are well-resolved from 194.73 THz to 195.76 THz (1531.46 nm to 1539.54 nm), showing a good agreement with the simulated spectra using the Hitran database.

### Performance analysis

Phase-sensitive detection, different from the Fourier transform in conventional DCS, is a powerful tool for filtering small sinusoidal signals out of random noise, even in extremely noisy environments. The QTF is a resonant sound transducer, which is very suitable for working with a phase-sensitive detector to complete the acoustic and electrical filtering in order. In the QEMR-PAS technique, the QTF acts as an audio filter with a narrow acoustic detection bandwidth Δ*f*_1_ (typically a few hertz), removing the acoustic noise outside of its detection bandwidth and extracting the resonant acoustic component from multiple heterodyne sound waves. The phase-sensitive detector after the QTF enables an extremely narrow electrical detection bandwidth Δ*f*_2_ (typically 0.1 Hz), further reducing the electronic noise. Thus, a high SNR is reached using the two-stage narrow bandwidth filtering in the processes of optical-acoustic and acoustic-electric conversions, respectively.

A background noise analysis of QEMR-PAS shows that two primary noise sources are the thermal noise associated with mechanical dissipation in the QTF and the thermal noise of the feedback resistor (see Supplementary Note S4). At the same time, the signal amplitude of QEMR-PAS is proportional to the optical power of the two teeth involved in the generation of the beat note, the *Q* factor of QTF, the concentration of target species, and inversely proportional to the QTF resonance frequency. Therefore, the SNR of QEMR-PAS can be expressed as:

7 $${SNR}\propto \frac{C}{\varDelta {f}_{1}}\frac{\sqrt{{P}_{{tooth}1}{P}_{{tooth}2}}}{{N}_{{th}}}$$

8 $${N}_{{th}}={R}_{g}\sqrt{\frac{4{k}_{B}T}{{R}_{{TF}}}}\sqrt{\varDelta {f}_{2}}$$

where *N*_*th*_ is the rms voltage noise at the pre-amplifier output, *C* is the concentration of the target species, *P*_*tooth*1_, *P*_*tooth*2_ are the optical powers of the two neighboring teeth of the two combs, *T* is the temperature, *R*_*TF*_ is the equivalent resistor of the QTF, *R*_*g*_ is the gain resistor of the pre-amplifier, and *k*_*B*_ is Boltzmann constant.

For conventional DCS, the SNR can be written as^1,15^:

9 $${SNR}\propto \frac{C}{M}\frac{\sqrt{{P}_{{comb}1}{P}_{{comb}2}}}{{NEP}}\sqrt{\tau }$$

where M is the number of comb teeth, ${P}_{{comb}1}$ and ${P}_{{comb}2}$ are the optical powers of the two combs, NEP is the photoreceiver’s noise equivalent power, and *τ* is the averaging time. By comparing Eqs. (7), (9), QEMR-PAS removes the averaging time τ, which is imposed by the Fourier transform for the full spectrum analysis. Instead, QEMR-PAS utilizes the integration time of the lock-in amplifier (LIA) for each tooth. However, it is important to note that this does not imply that QEMR-PAS achieves a faster acquisition time. In fact, in the acoustic frequency domain, the acquisition time of QEMR-PAS is longer compared to a RF dual-comb spectrometer due to its lower frequency^14,24^. Furthermore, the QTF with a high Q value results in a longer accumulation time. The longer acquisition times represent a trade-off amongst the dynamic range, sensitivity, and detection cost. Moreover, Hertz/sub-Hertz levels of detection bandwidths in acoustics (Δ*f*_1_) and electronics (Δ*f*_2_) further improve the QEMR-PAS SNR. In conventional DCS, the shot-noise-limited SNR does not apply, but the photoreceiver’s NEP dominates, according to Eq. (9). Clearly, the route to a higher SNR for conventional DCS is the longer averaging time and the lower NEP.

For a comparison, the Eq. (9) can be rewritten as:

10 $${SNR}\propto \frac{C}{M}\left(\frac{\sqrt{{P}_{{comb}1}{P}_{{comb}2}}}{{NEP}\sqrt{\varDelta {f}_{2}}}\right)\sqrt{\varDelta {f}_{2}}\sqrt{\tau }$$

Although there is the same factor $\sqrt{{P}_{{toot}h1}{P}_{{toot}h2}}=\sqrt{{P}_{{comb}1}{P}_{{comb}2}}/\sqrt{{M}^{2}}$ in Eqs. (7), (10), their range of values is significantly different. In conventional DCS, the high peak intensity of the frequency comb—the temporal nature of the ultrashort pulse—does not bring any advantages but limits the dynamic range of the interferometric signal by saturating a photoreceiver or a digitizer. For example, a commercial 100 MHz InGaAs amplified photoreceiver might have a limited $\sqrt{{P}_{{comb}1}{P}_{{comb}2}}/({NEP}\cdot \sqrt{\varDelta {f}_{2}})$ of ∼500 (given by the ratio of the maximum peak power to the NEP integrated over a detection bandwidth of $\varDelta {f}_{2}$), which limits further SNR improvement in conventional DCS. Conversely, QEMR-PAS can benefit from the high optical power levels for comb. A previous work^32^ demonstrated that with a 1.4 W optical power excitation from a single mode CW laser emitting at 1560 nm, a detection sensitivity of ppb level can be achieved in quartz-enhanced photoacoustic spectroscopy without saturation behavior, thanks to a super-large linear dynamic range of QTF. This means that a tooth power $\sqrt{{P}_{{toot}h1}{P}_{{toot}h2}}$ in QEMR-PAS can be set up to 1.4 W further to improve the SNR.

Differential phase noise between the combs is not involved in Eqs. (7), (9). Even in mutually coherent combs, a residual phase noise pedestal exists on any given tooth. Phase noise at Fourier frequencies below the tooth spacing will contribute to slow baseline variations, and those above tooth spacing will affect neighboring comb teeth, limiting the SNR. In our experiment, an active real-time phase correction was employed, suppressing the differential phase noise (see Supplementary Note S3).

**Fig. 6:**
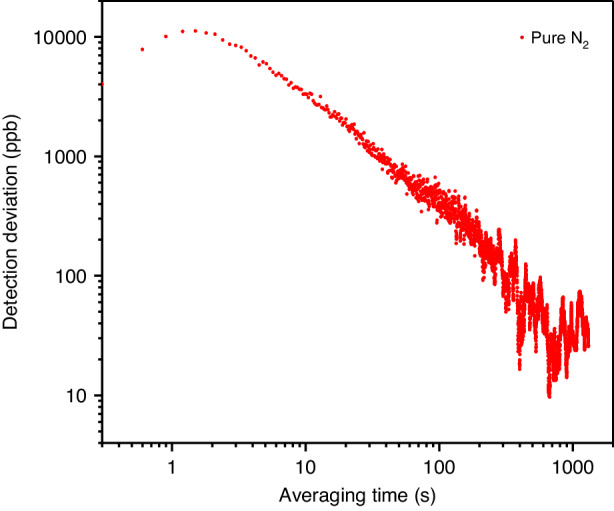
Allan variance stability analysis of the QEMR-PAS system. The red data point represents the Allan variance of the QEMR-PAS system when operating with pure N_2_

To assess the long-term stability of the QEMR-PAS system, an Allan variance analysis for the main comb tooth component (*n* = 0) was conducted with pure N_2_ while varying the averaging time. The results depicted in Fig. 6 reveal that the sensitivity of the QEMR-PAS system can be enhanced by extending the averaging time. Specifically, an optimal detection limit of 50 ppb was achieved with an averaging time of 316 s.

A side-by-side comparison of performance for conventional DCS, dual-frequency comb photoacoustic spectroscopy (DC-PAS) based on a microphone and QEMR-PAS is shown in Table 1. For conventional DCS, two types of photoreceivers, InGaAs for NIR-DCS and HgCdTe for MIR-DCS, are considered for a spectral coverage of 0.8 μm-1.6 μm and 3 μm-10 μm, respectively. However, QEMR-PAS and DC-PAS offer wavelength-independent detectors, enabling spectral detection across a wide range from UV to THz. This is achieved by detecting sound wave rather than relying on optical waves. For quantitative measurements, in cases where suitable photoreceivers are unavailable for a specific wavelength range, the reference signal could be alternatively implemented through the photoacoustic detection of black-body absorption to measure the intensity of light. Furthermore, the QEMR-PAS technique has a dynamic range of 63 dB with a spectral resolution of 43 MHz, which is several orders of magnitude better than that of NIR-DCS, MIR-DCS and DC-PAS. The total integration time is determined by multiplying 0.3 s by the number of combs, which results in 9.6 s. The predicted noise (*N*_*th*_) of QEMR-PAS is calculated to be 2.40 μV when substituting *T* = 300 K, *R*_*g*_ = 10 MΩ, *R*_*TF*_ = 120 kΩ, Δ*f*_2_ = 0.417 Hz into Eq. (8). The predicted value is in excellent agreement with the experimental value of 2.85 μV. Such a noise level results in a noise equivalent absorption (NEA) of 5.99 × 10^−6^ cm^-1^·Hz^-1/2^, which is one order of magnitude better than DC-PAS and same order as NIR-PAS and MIR-PAS. The corresponding normalized noise equivalent absorption coefficient (NNEA) is 5.99 × 10^−8^ cm^-1^·W·Hz^-1/2^.

**Table 1:** Side-by-side performance comparison between conventional DCS and QEMR-PAS

Techniques	Detector Type	Spectral coverage of detector (μm)	Dyna-mic range (dB)	Spectral resolution (GHz)	Avera-ging time (s)	Detection limit (ppm)	NEA (cm^−1^·Hz^-1/2^)
NIR-DCS^38^	InGaAs	0.8-1.6	27	1	0.0025	C_2_H_2_: 364	3.39 × 10^−6^
MIR-DCS^39^	HgCdTe	3–10	22	0.5	2	Mixture: -	6.40 × 10^−6^
DC-PAS^24^	Microph-one	All band	45	1	1000	C_2_H_2_: 10	2.90 × 10^−5^
QEMR-PAS	QTF	All band	60	0.043	9.6	C_2_H_2_: 4	5.99 × 10^−6^

## Discussion

In summary, extending DCS to broadband wavelength coverage with a large dynamic range and a high spectral resolution requires getting rid of the constraints of the principle and device of conventional DCS. Here we demonstrated the QEMR-PAS technique, in which a QTF selectively detects an audio frequency component of multiple heterodyne sound waves generated by a dual comb through the photoacoustic effect. Hence, the QEMR-PAS technique eliminates the need for wavelength-dependent photoreceivers and reduces computational complexity of Fourier transforms in conventional DCS. Instead, a miniature QTF and a simple phase-sensitive detector were employed as a sound transducer and a narrow band filter, which enables the development of a compact, low-cost sensor based on QEMR-PAS. Furthermore, the QEMR-PAS technique provides a wavelength-independent DCS detection method. The results show that this new technique is capable of providing an ultra-large dynamic range of 63 dB and ultra-narrow spectral resolution of 43 MHz (or ~0.3 pm). In terms of NEA, there is only an order of magnitude improvement over DC-PAS. But we still have a 20-dB reserve in dynamic range. The technological developments of optical frequency combs with higher average power (high pulse energies), up to watt level^35,36^, will bring huge benefits for QEMR-PAS, which is expected to lead to further improvement in detection sensitivity through the use of high power optical frequency comb in the near future.

It is worth noting that while utilizing a QTF offers several advantages, including wavelength-independent response, large dynamic range, and high sensitivity, it also results in a longer acquisition time for the system due to the high Q value of the QTF. In future studies, the proposed QEMR-PAS technique can be combined with beat frequency detection^27^ to effectively reduce the system’s acquisition time. Meanwhile, while the current demonstration of the QEMR-PAS system relies on electro-optic combs, we firmly believe that QEMR-PAS holds great promise for application with mid-infrared combs for stronger gas absorption. This can be achieved by utilizing mid-infrared electro-optic frequency comb^37^ and employing custom acousto-optic shifters based on germanium.

## Materials and methods

### Dual-comb source

A free-running CW laser is used to generate a dual comb. The optical frequency shifts ${f}_{\mathrm{0,1}}(t)$ and ${f}_{\mathrm{0,2}}$ are provided by two acousto-optic modulators, respectively. ${f}_{\mathrm{0,1}}(t)$ varied from 80 MHz + $\frac{1}{2}{f}_{Q}$ to 80 MHz + $\frac{3}{2}{f}_{Q}$ and ${f}_{\mathrm{0,2}}\,$= 80 MHz. The modulate frequencies of two electro-optic modulations are ${f}_{{rep},1}\,$= 1 GHz and ${f}_{{rep},2}\,$= 1 GHz + $\frac{{f}_{Q}}{N}\,$Hz, where ${f}_{Q}$ represents the resonance frequency of the QTF, *N* represents the preset number of comb teeth. All modulation sources are synchronized together to a 10 MHz frequency standard to ensure mutual-coherence in the modulation process.

### QEMR-PAS system

The comb light passes through the gas chamber and the QTF. A custom QTF (${f}_{Q}$ = 15849.4 Hz) is used to realize QEMR-PAS. The electrical signal output is introduced into a pre-amplifier, then demodulated by LIA-2. LIA-1 is used to demodulate the reference signal from the photoreceiver. The phase fluctuations of the system are given by LIA-1 and used to synchronize with LIA-2. The normalized absorption spectrum is given by the ratio of the R value of LIA-2 to that of LIA-1. The final signal value of each comb tooth is averaged over the acquisition time.

### Calculation of NNEA

The NNEA coefficient can be determined by the formula $\frac{P}{N}\cdot {\alpha }_{\min }/\sqrt{\triangle {f}_{2}}$. Here, *P* represents the total power of the combs, and *N* is the number of spectral lines, yielding the average power for each spectral line (10 mW). The minimum detectable absorption coefficient, ${\alpha }_{\min }$ is calculated as 4.6 $\times$10^-6^ cm^-1^, sourced from the HITRAN database, when the absorption wavelength of the target trace gas and the detection limit of the sensor are known. The electrical filter bandwidth $\triangle {f}_{2}$ is determined as 0.59 Hz when the time constant and filter slope of the LIA-2 is set as 300 ms and 12 dB, respectively.

### Supplementary information

Supplementary information for Quartz-enhanced multiheterodyne resonant photoacoustic spectroscopy

## Data Availability

The authors declare that all data supporting the findings of this study can be found within the paper and its Supplementary information files. Additional data supporting the findings of this study are available from the corresponding author (L.D.) upon reasonable request.
