# Supplementary material for: Quartz-enhanced multiheterodyne resonant photoacoustic spectroscopy
Source: Light Sci Appl. 2024 Mar 22;13:77. doi: 10.1038/s41377-024-01425-1 (PMC10957990; doi:10.1038/s41377-024-01425-1)
Supplement: Supplementary file 1 — Supplementary information for Quartz-enhanced multiheterodyne resonant photoacoustic spectroscopy [file 41377_2024_1425_MOESM1_ESM.docx]

Supplementary information for

Quartz-enhanced multiheterodyne resonant photoacoustic spectroscopy

**Jiapeng Wang^1,2,4^, Hongpeng Wu^1,2,4^, Angelo Sampaolo^3^, Pietro Patimisco^3^, Vincenzo Spagnolo^1,3^, Suotang Jia^1,2^ and Lei Dong^1,2,*^**

^1^State Key Laboratory of Quantum Optics and Quantum Optics Devices, Institute of Laser Spectroscopy, Shanxi University, Taiyuan 030006, China.

^2^Collaborative Innovation Center of Extreme Optics, Shanxi University, Taiyuan 030006, China.

^3^PolySense Lab, Dipartimento Interateneo di Fisica, University and Politecnico of Bari, CNR-IFN, Via Amendola 173, Bari 70126, Italy.

^4^These authors contributed equally to this work.

[*donglei@sxu.edu.cn](mailto:*donglei@sxu.edu.cn)

Supplementary Note S1: Dynamic range of quartz tuning forks

To assess the linear dynamic range of the quartz tuning fork (QTF), an electric excitation method is used to simulate the sound wave excitation of the QTF. A sinusoidal voltage signal is introduced into one of the two QTF pins, exciting the QTF prongs to vibrate through the photoacoustic effect. The piezo-electric current produced by the QTF deformation is detected from another pin of the QTF. The dynamic range of the QTF is shown in Fig. **S1**. A QTF sensitivity of 27.3 μV/μPa and a reference sound intensity of 10^-12^ W/m^2^ are adopted here. The sound intensity is given by $I=P^{2}/\rho c$, where $\rho$ is the density of the air medium, *P* is the acoustic pressure and *c* is the speed of the sound. The results show that a linear dynamic range covers over six orders of magnitude from thermal noise, corresponding to a sound intensity of -17 dB, to the QTF’s breakdown deformation, corresponding to a sound intensity of 46 dB. It covers a linear dynamic range over six orders of magnitude. In quartz-enhanced multiheterodyne resonant photoacoustic spectroscopy (QEMR-PAS), an actual dynamic range of >40 dB is observed during the measurement of photoacoustic signals. There is still a dynamic reserve of 20 dB for the stronger photoacoustic signal. The QTF operates at its resonant frequency for all the measurements.

The QTF sensitivity of 27.3 μV/μPa is obtained by theoretically analyzing a typical QTF-based photoacoustic process as follows. Specifically, the approximate solution of the acoustic pressure produced by a laser beam in unbounded space is given by ^[1,2]^

| $P(r,t)\approx A[J_{0}(\frac{\omega r}{c})\cos(\omega t)+Y_{0}(\frac{\omega r}{c})\sin(\omega t)]$ | (S1) |
| --- | --- |

Here *J*_0_ and *Y*_0_ are the zeroth-order Bessel functions of the first and second kinds, respectively, $\omega$ is the resonant angular frequency, and *r* is the radial distance from the axis of the laser beam. *A* is given by

| $A=(\gamma-1)\omega\kappa\frac{P}{8c^{2}}$ | (S2) |
| --- | --- |

where $\gamma$ is the adiabatic coefficient of the specie, $\kappa$ is the effective absorption coefficient, and *P* is the laser’s optical power. The force density $F_{tine}(t)$ on tines is given by the acoustic pressure difference between the inner *P_i_* and outer surfaces *P_o_* of the tines multiplied by the thickness of the tines *d*, namely


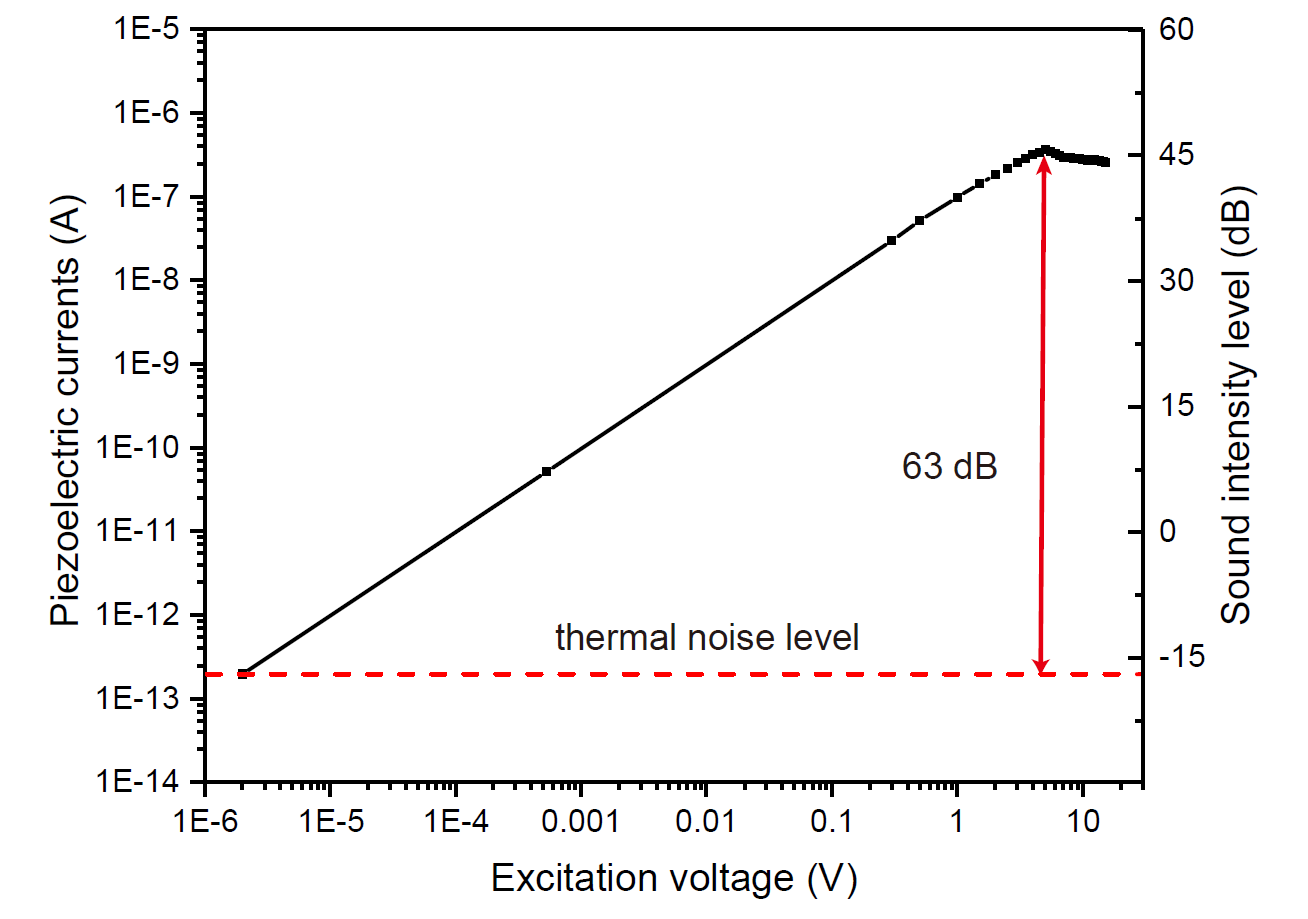


**Figure S1. Liner dynamic range of the QTF.** The horizontal axis shows the voltage imposed on one of two QTF pins to simulate the sound pressure excitation. The piezoelectric currents excited by the simulated sound pressure are shown on the left vertical axis. The relationship between the piezoelectric output and the sound intensity is given theoretically by analyzing a typical QTF-based photoacoustic process.

| $F_{tine}(t)=2d\left[ P_{i}(r_{i},t)-P_{o}(r_{o},t) \right]$ | (S3) |
| --- | --- |

The pressure difference between the inner and outer surfaces, *P_i_* and *P_o_*, is converted into an electric charge through the piezoelectric effect. Meanwhile, the displacement of the vibrating arm of the QTF can be described by the classical harmonic oscillator model. The displacement of the tuning fork tine *x*(*t*) is given by

| $x(t)=\frac{F_{tine}(t)}{\omega}/\sqrt{1-\frac{1}{4Q^{2}}}e^{i\omega t}$ | (S4) |
| --- | --- |

*Q* is the quality factor of the QTF, and the current generated by the piezoelectric effect is determined by

| $I=2\alpha\frac{dx}{dt}$ | (S5) |
| --- | --- |

and $\alpha$ is given by

| $\alpha=\sqrt{\frac{m_{e}\omega}{2QR}}$ | (S6) |
| --- | --- |

where *m_e_* represents the effective mass of the QTF, and *R* is the equivalent resistor of the QTF.

The sound pressure *P_i_* is 80.2 μPa and *P_o_* is 69.2 μPa, and the corresponding piezoelectric current *I* is 30.7 pA after all the parameters are substituted into the above equations. The piezoelectric current is converted to 0.3 mV by a transimpedance pre-amplifier with a gain resistor of 10 MΩ. Therefore, the sound pressure sensitivity of the QTF is 27.3 μV/μPa.

Supplementary Note S2: QTF geometry and its fabrication process

Custom QTFs for QEMR-PAS are designed and fabricated. The prong length *l*, width *w*, thickness *t*, and prong spacing of the QTF are 9.4 mm, 2 mm, 0.25 mm, and 0.8 mm, respectively, as shown in Fig. **S2**. The QTF geometry determines a resonant frequency of ~15.8 kHz. Such a resonant frequency is an optimal value, considering vibrational-translational relaxation rate levels of most gas molecules and environmental noise suppression. A z-cut quartz wafer with a 2° rotation along the x-axis, which can provide thermally stable modal frequencies of flexural vibration, is selected for the realization of custom QTFs. Standard photolithographic techniques are used to etch the QTFs. A three-dimensional crystal structure is generated by chemical etching in a hydrogen fluoride solution. Then 450$\overset{o}{A}$ /2500 $\overset{o}{A}$ Cr/Au thin layer is deposited on both sides of the QTF using shadow masks, to collect the charge generated by the piezoelectric effect of the QTF.


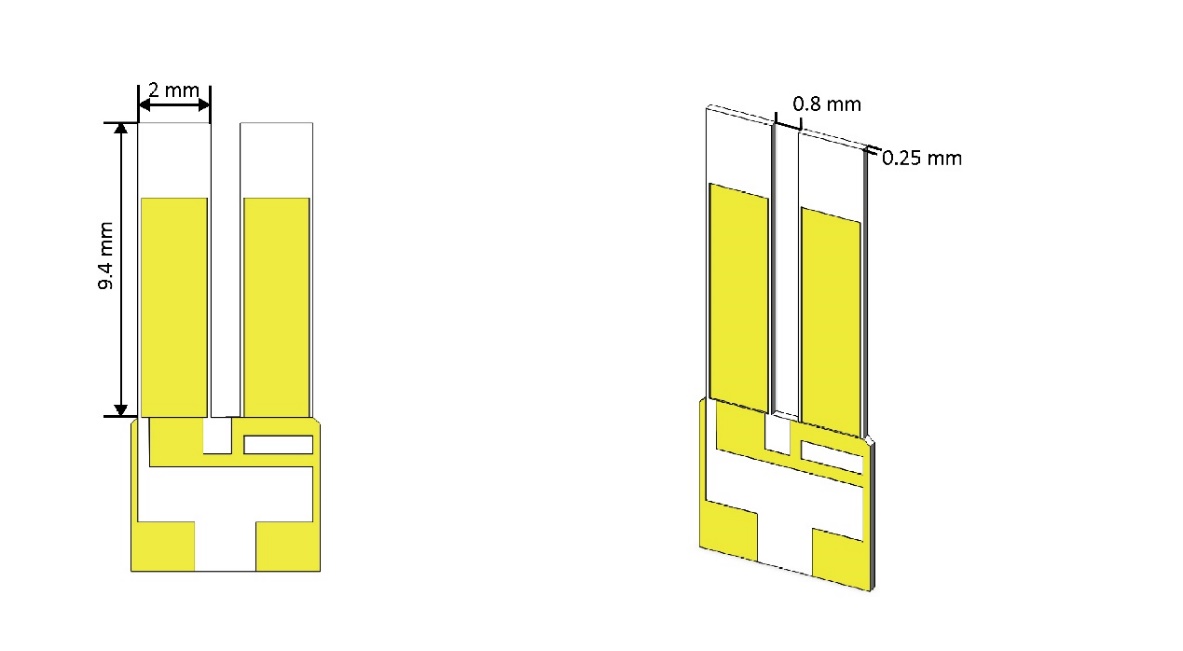


**Figure S2. The structure of the custom QTF.** Schematic of the geometrical dimensions of the custom tuning fork. The yellow areas represent the section of electrode layout.

Supplementary Note S3: Active real-time phase correction

The phase fluctuation caused by the asymmetric optical paths will affect the signal-to-noise ratio (SNR), especially for weak signal detection. In our experiment, an active real-time phase correction method is adopted to suppress the phase fluctuation, as shown in Fig. **S3**.


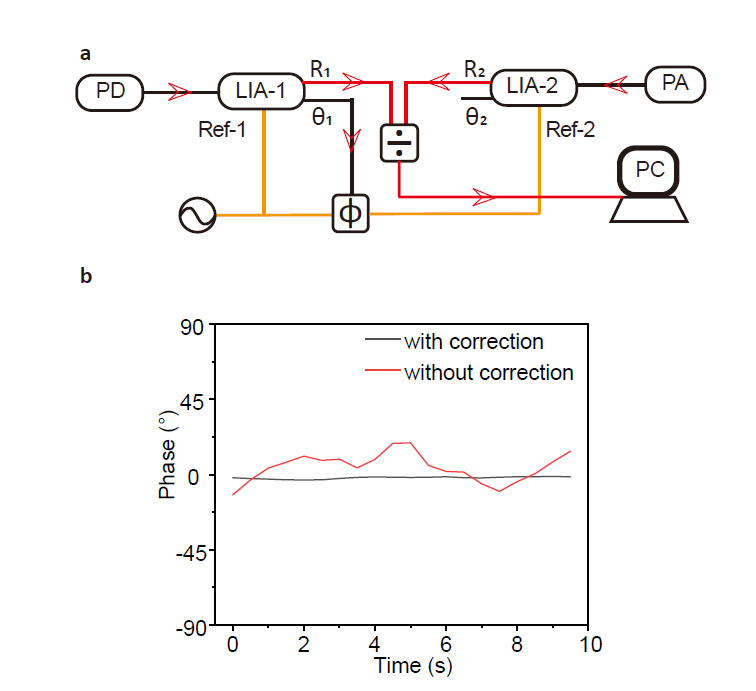


**Figure S3. Active real-time phase correction method. a** Schematic of the phase correction system. An oscillation source produces a sinusoidal signal of the same frequency as the QTF, as the reference signal Ref-1. The phase fluctuation θ_1_ of the measured comb tooth with respect to Ref-1, is obtained from the photodetector (PD) output by a quadrature lock-in amplifier (LIA-1). Ref-1 is phase shifted by θ_1_ using a phase shifter and then introduced into another quadrature lock-in amplifier (LIA-2) as the reference signal Ref-2. The amplitude R_1_ output by LIA-1 is used to normalize the QEMR-PAS signal. **b** Phase fluctuation θ_2_ of the QEMR-PAS signal with and without correction. After correction, the phase fluctuation is well suppressed.

An oscillation source generates a sinusoidal signal Ref-1, which has the same frequency as the QTF. The photodetector (PD) signal output is demodulated with respect to Ref-1 by a quadrature lock-in amplifier (LIA-1). The change in the phase θ_1_ obtained from LIA-1 reflects the phase fluctuation of the measured comb tooth with respect to Ref-1, which is caused by the asymmetric optical paths. Since the photoacoustic heterodyne signals from the pre-amplifier (PA) are demodulated by another quadrature lock-in amplifier (LIA-2), Ref-1 is phase shifted by θ_1_ as the reference signal Ref-2 of LIA-2. In this way, the phase fluctuation can be corrected effectively in real-time. The results show that the standard variance of the phase fluctuation decrease from 44 mπ without correction (in red) to 3.6 mπ with correction (in black) in an observation time of 10 s. Furthermore, the amplitude R_1_ obtained from LIA-1 reflects the intensity of the measured comb tooth. Hence, the QEMR-PAS signal R_2_ from LIA-2 should be normalized to R_1_ to yield the normalized photoacoustic spectrum.

Supplementary Note S4: Background noise analysis of QEMR-PAS

Our QEMR-PAS experiments utilize a transimpedance pre-amplifier (PA), as shown in Fig. **S4**, to obtain the QTF electrical response. The QTF noise in this circuit, measured at the amplifier output at the resonant frequency, is equal to the thermal noise of the equivalent resistor *R*

| $N_{th}=R_{g}\sqrt{\frac{4k_{B}T}{R_{TF}}}\sqrt{\Delta f_{2}}$ | (S7) |
| --- | --- |

where $\Delta f_{2}$ is the electrical bandwidth of the phase-sensitive detection, *R_g_* is the gain resistor of the pre-amplifier, *R_TF_* is the equivalent resistor of the QTF, *k_B_* is the Boltzmann constant and *T* is the QTF temperature. A gain resistor *R_g_* = 10 MΩ is used. The gain resistor *R_g_* also introduces noise with a spectral density $\sqrt{4k_{B}TR_{g}}$, but it is $\sqrt{R_{g}/R_{TF}}$ times lower than the QTF noise and can be usually neglected for typical values of *R_TF_* ~ 10-200 k$\Omega$.


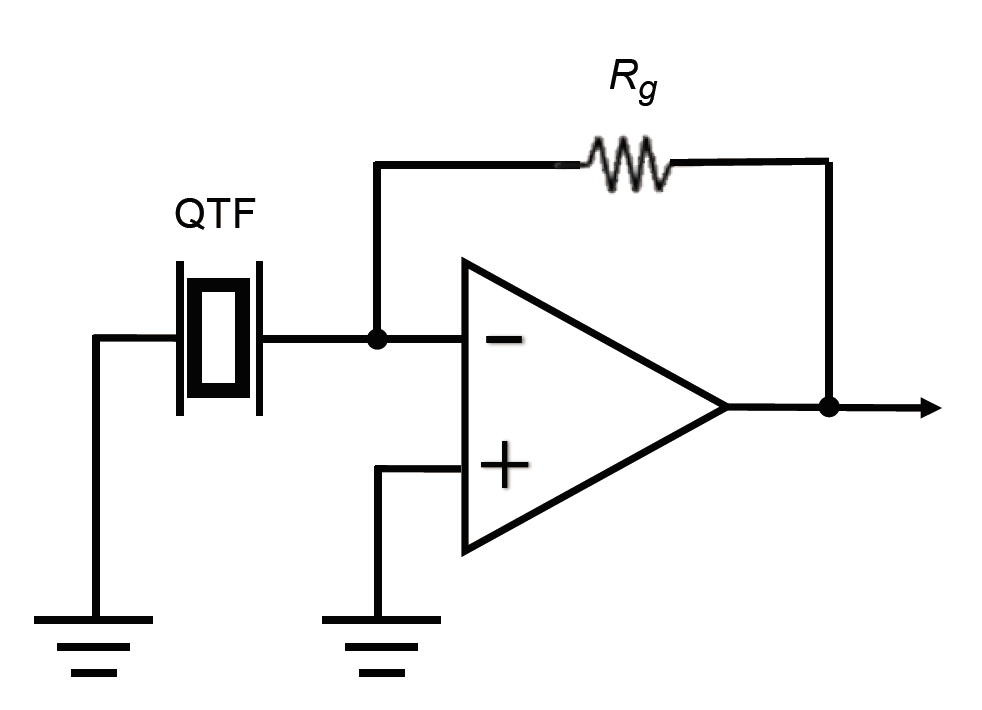


**Figure S4.** **Schematic of the QTF signal detection for QEMR-PAS.** Two primary noise sources are the thermal noise associated with mechanical dissipation in the QTF and the thermal noise of the feedback resistor.

References

[1] Petra, N., Zweck, J., Kosterev, A. A., Minkoff, S. E. & Thomazy, D. Theoretical analysis of a quartz-enhanced photoacoustic spectroscopy sensor. *Applied. Physics. B 94, 673-680, (2009).*

[2] Miklós, A., Hess, P. & Bozóki, Z. Application of acoustic resonators in photoacoustic trace gas analysis and metrology. *Review of Scientific Instruments.* **72**, 1937-1955, (2001).
